# Urinary incontinence and sleep complaints in community dwelling older adults

**DOI:** 10.5935/1984-0063.20180020

**Published:** 2018

**Authors:** Neda Sadat Nazaripanah, Yadollah Abolfathi Momtaz, Farideh Mokhtari, Robab Sahaf

**Affiliations:** 1 Iranian Research Center on Aging, University of Social Welfare and Rehabilitation Sciences, Tehran, Iran, Gerontology - Tehran - Tehran - Iran.; 2 Student Research Committee, University of Social Welfare and Rehabilitation Sciences, Tehran, Iran, Gerontology - Tehran - Tehran - Iran.; 3 Malaysian Research Institute on Ageing (MyAgeing), Universiti Putra Malaysia, Gerontology - Serdang - Selangor - Malaysia.

**Keywords:** Aged, Community Dwelling, Sleep Wake Disorders, Urinary Incontinence

## Abstract

Sleep disorder is associated with poor quality of life in old age. Therefore, it is imperative to identify contributing factors leading to sleep disorder. The current study aimed to examine the impact of urinary incontinence on sleep complaint after controlling for potential sociodemographic and health covariates. **Materials and Methods:** A cross-sectional study was conducted on a sample of 184 community dwelling older adults 60 years and older in Yazd, Iran, 2016. In order to obtain the sample a multistage proportional random sampling technique was employed. Sociodemographic characteristics, sleep complaint, and urinary incontinence were collected from medical records. Statistical analyses were performed using SPSS version 24. A multiple logistic regression analysis was used to examine the impact of urinary incontinence on sleep complaint after controlling for potential covariates. **Findings:** A total of 184 respondents with a mean age of 68.48±6.65 years (age range, 60-87 years) were included in the study. About 70% of the respondents were women, 72.8% were married, 68.5% were not formally educated, and 21.7% were living alone. The prevalence of sleep complaint and urinary incontinence were 27.2% (95% CI: 21-34) and 22.3% (95% CI: 17-29), respectively. The results of the multiple logistic regression analysis revealed respondents with urinary incontinence were four times more likely to suffer from sleep complaint than those without urinary incontinence after adjusting for potential covariates (AOR=4.04, 95% CI: 1.74-9.35, *p*<0.001). **Conclusion:** Based on the results of this present study, which showed that urinary incontinence independently contributed to sleep complaint among older adults, it is necessary to employ effective interventions for controlling urinary incontinence to reduce sleep complaints.

## INTRODUCTION

It has been widely documented that sleep disorders are more prevalent in old age and lead to poor quality of life and serious complications^1^. The world’s population is ageing rapidly^2^. It is estimated that more than one-fourth of the global population will be 60 years and older by 2100^3^. Like other countries around the world, Iran is experiencing the population aging phenomenon, due to declining fertility rates combined with increasing life expectancy. According to the latest census taken in 2016, aged population (60 years and older) accounts for 9.28% of the Iranian population^4^. Moreover, the percentage of the elderly population is predicted to reach 30% in Iran by 2050^3^.

In light of this demographic change, the World Health Organization has emphasized measures to help older people attain successful aging and the best possible quality of life^5^, therefore, improving and maintaining quality of life in aging population is a major priority for policymakers^6^^,^^7^.

Sleep is one of the most important factors that can contribute to physical and mental health, quality of life, and the well-being of people in all stages of life, especially in old age^8^^,^^9^. Considering the importance of sleep in older adults’ quality of life, several gerontological studies have conducted to find influencing factors that could contribute to sleep disorders^8^. Sleep disorder is one of the most commonly problems in the aging population and affects around 50% of the elderly population^10^.

According to the National Health Survey conducted in five provinces of Iran, approximately 45% of the elderly had sleep disorders^11^. Sleep disorder in the elderly can lead to reduced daily functioning, more frequent mental disorders, declined quality of life, increased risk of fall, and increased mortality rate^8^^,^^12^. Several predictors such as physical, psychosocial, and environmental factors may be contributed to sleep disorder^8^. Additionally, sleep disorder in the elderly may be attributed to various factors, including age-dependent intrinsic lightening of sleep homeostatic processes^13^^,^^14^, chronobiological changes^15^, increased prevalence of medical conditions, increased drug intake, and lifestyle changes^16^. Moreover, it has been documented that age, sex, level of education, and living arrangement, can be associated with sleep disorder^17^^,^^18^.

One of the geriatric issues that may lead to sleep disorder is urinary incontinence. However, limited studies have examined its impact on sleep disorder^10^^,^^19^. According to Hazard, urinary incontinence can be defined as any involuntary leakage of urine, whether temporarily or permanently^10^. It is a common problem in the elderly, as it increases with age and affects about 35% and 22% of elderly women and men, respectively^10^^,^^20^^,^^21^.

Urinary incontinence may disrupt sleep patterns and daily functioning^22^. According to a study of the elderly in Australia, urinary incontinence was associated with nocturnal sleep disorder and daily sleepiness^19^. Therefore, this present study aimed to investigate the relationship between urinary incontinence and sleep complaint in the elderly population after controlling for potential covariates.

## MATERIALS AND METHODS

This cross-sectional study was conducted to examine the relationship between urinary incontinence and sleep complaint among 184 elderly individuals who were selected via multistage proportional random sampling technique in 2016. For sample collection, in the first stage, one healthcare center was selected among healthcare centers from four districts of Yazd, Iran (this area included a total of four centers). In the second stage, considering the population percentage covered by each healthcare center, medical records of the subjects were randomly selected. All necessary information was extracted from the medical records. In cases of incomplete information, the individual was contacted.

### Study setting

The current study was conducted in Yazd, one of the historical cities in central Iran, which was registered in the UNESCO World Heritage List in 2017. According to the latest census taken in 2016, the population of Yazd is 611,466. Approximately eight percent of the population of Yazd are 60 years and older^4^.

### Measures

*Demographic questionnaire*

Demographic data, including age, gender, marital status, level of education, and living arrangement were extracted from medical records.

*Covariates*

On the basis of previous work in examining risk factors affecting sleep disorders, several health-related covariates including hypertension^23^, diabetes^24^, vision problems^25^, hearing impairment^26^, and myocardial infarction^1^ were selected.

*Body mass index (BMI)*

Weight was measured barefoot with minimum clothing using a digital scale (Seca; precision, 100 g). Height was measured with a stadiometer (precision, 0.5 cm) without shoes or a hat in a standing position with heels touching the ground. Finally, BMI was calculated by dividing weight by squared height in units of kg/m^2^.

*Sleep complaints*

Subjective sleep complaint as the dependent variable was ascertained from binary responses to the following items: “early morning awakening”, “late night sleeping”, “difficulty falling asleep”, and “nocturnal awakening”. A positive response to any items was categorized as presence of sleep complaint.

*Urinary incontinence*

Urinary incontinence was assessed based on six criteria, including “frequent urination”, “waking up to urinate during night”, “blocked or reduced flow of urine and reflowing”, “sudden, intense urge to urinate, followed by an involuntary loss of urine”, “urine leakage by coughing, sneezing, laughing, and rising from the ground”, and “stress test”. There was no need for a stress test if he/she had urine leakage in coughing, sneezing, laughing, or rising from the ground. Otherwise, he/she was asked to undergo the stress test. If he/she needed to urinate, he/she stood with feet shoulder-width apart, while kneeling slightly and relaxing the pelvic floor; then, he/she was asked to cough strongly. If he/she confirmed sudden urine leakage after coughing, the stress test was positive. If he/she had at least one symptom or positive stress test, he/she was identified as having urinary incontinence^27^.

*Data analysis*

Collected data were statistically analyzed in terms of missing values and outliers, and then were tested for a normal distribution. Statistical analyses were performed using SPSS version 24. Chi square was used to determine the relationship between sleep complaint and categorical variables. Additionally, independent *t*-test was employed to find out relationship between sleep complaint and continues variables. Moreover, multiple logistic regression analysis was used to determine the relationship between urinary incontinence and sleep complaint after adjusting for potential covariates. The possible sociodemographic and health characteristics were chosen according to Greenland’s entry criteria for statistical modelling^28^.

*Findings*

Demographic and health-related characteristics of the study participants are presented in Table 1. The respondents were included a total of 184 community dwelling older adults 60 years and older with a mean age of 68.48±6.65 years (age range, 60-87 years). Approximately 70% of the respondents were women, 72.8% were married, 68.5% were not formally educated, and 21.7% were living alone.

**Table 1:** Profile of the study sample.

Variable	Category	n	%
Sex	Female	129	70.1
	Male	55	29.9
Level of Education	Formal Education	58	31.5
	No Formal Education	126	68.5
Marital status	Unmarried	50	27.2
	Married	134	72.8
Living Arrangement	Alone	40	21.7
	Non-alone	144	78.3
Hypertension	Yes	87	47.3
	No	97	52.7
Myocardial Infarction	Yes	23	12.5
	No	161	87.5
Diabetes	Yes	57	31
	No	127	69
Vision Problems	Yes	82	44.6
	No	102	55.4
Hearing Impairment	Yes	27	14.7
	No	157	85.3
Urinary Incontinence	Yes	41	22.3
	No	143	77.7
Sleep complaint	Yes	50	27.2
	No	134	72.8

As shown in Table 1, a history of hypertension (47.3%) showed the highest prevalence, while history of myocardial infarction (12.5%) showed the lowest prevalence. The mean BMI was 26.55±4.79 kg/m^2^. Based on these findings, the prevalence of sleep complaint and urinary incontinence were 27.2% and 22.3%, respectively.

A series of Chi-square tests were used to determine the relationship between sleep complaint and both demographic and health-related variables. As can be seen from Table 2, a significant association was found between sleep complaint with sex (χ^2^=4.63, *p*<0.05), history of hypertension (χ^2^=7.7, *p*<0.01), vision problems (χ^2^=17.98, *p*<0.001), and urinary incontinence (χ^2^=22.3, *p*<0.001). More sleep complaint was observed among women, older adults with hypertension, vision problems, and urinary incontinence (Table 2). By contrast, no significant associations were observed between sleep complaint and family status, level of education, marital status, history of myocardial infarction, diabetes, and hearing impairment (Table 2).

**Table 2:** Characteristics of the study sample by sleep complaint.

Variable	Category	Sleep complaint				χ2	*p*-value
		Yes		No			
		n	%	n	%		
Sex	Female	41	31.8	88	68.2	4.63	0.031
	Male	9	16.4	46	83.6		
Level of Education	Formal Education	16	27.6	42	72.4	0.01	0.932
	No Formal Education	34	27	92	73		
Marital status	Unmarried	14	28	36	72	0.02	0.878
	Married	36	26.9	98	73.1		
Living Arrangement	Alone	11	27.5	29	72.5	0.01	0.958
	Non-alone	39	27.1	105	72.9		
Hypertension	Yes	32	36.8	55	63.2	7.7	0.006
	No	18	18.6	79	81.4		
Myocardial Infarction	Yes	10	43.5	13	56.5	3.53	0.060
	No	40	24.8	121	75.2		
Diabetes	Yes	20	35.1	37	64.9	2.61	0.106
	No	30	23.6	97	76.4		
Vision Problems	Yes	35	42.7	47	57.3	17.98	0.001
	No	15	14.7	87	85.3		
Hearing Impairment	Yes	11	40.7	16	59.3	2.94	0.086
	No	39	24.8	118	75.2		
Urinary Incontinence	Yes	23	56.1	18	43.9	22.3	0.001
	No	27	18.9	116	81.1		

A series of independent *t-*tests were employed to compare groups of respondents with and without sleep complaint in terms of age and BMI. A significant difference was found between respondents with sleep complaint and those without sleep complaint in terms of BMI (t (182) =-2.38; *p*<0.05), wherein the mean BMI in people with sleep complaint (M=27.91, SD= 5.48) was significantly higher than that of those without sleep complaint (M=26.04, SD=4.42). Nevertheless, no significant difference was detected in the mean age of subjects with sleep complaint (M=67.28, SD=5.67) *versus* those without sleep complaint (M=68.93, SD=6.95) (t (182) =1.50, *p*=0.134).

As the main purpose of this study was to examine the impact of urinary incontinence on sleep complaint after controlling for potential sociodemographic and health covariates, a multiple logistic regression analysis was used. The Hosmer-Lemeshow goodness-of-fit test showed that the multiple logistic regression model fit the observed data accurately (χ^2^ (8)=10.09; *p*=.259).

As depicted in Table 3, the results of the multiple logistic regression analysis showed that the full model was statistically significant (χ^2^=174.87, *p*<0.001) and urinary incontinence (AOR=4.04, 95% CI: 1.74-9.35, *p*<0.001) is significantly contributed to sleep complaint after adjusting for potential covariates including age, sex, history of hypertension, myocardial infarction, diabetes, vision problems, hearing impairment, and BMI. The adjusted logistic regression analysis revealed respondents with urinary incontinence were four times more likely to suffer from sleep complaint than those without urinary incontinence.

**Table 3:** The results of multiple logistic regression analysis to predict sleep complaint.

				95% C.I. for EXP(B)	
Variable	B	*p*-value	AOR	Lower	Upper
Age	-0.05	0.139	0.95	0.89	1.02
Sex	-0.62	0.202	0.54	0.21	1.39
Hypertension	0.72	0.088	2.06	0.90	4.74
Myocardial Infarction	0.46	0.402	1.59	0.54	4.66
Diabetes	-0.30	0.496	0.74	0.32	1.75
BMI	0.00	0.923	1.00	0.92	1.08
Vision Problems	1.03	0.010	2.80	1.27	6.17
Hearing Impairment	0.46	0.375	1.58	0.57	4.35
Urinary Incontinence	1.39	0.001	4.03	1.74	9.35

BMI: Body Mass Index AOR: Adjusted Odds Ratio, Sex coded as Male=1, Female=0 Hosmer-Lemeshow, χ^2^ (8) =10.09; *p*=.259 Log likelihood: χ^2^ = 174.87, *p*<0.001

## DISCUSSION

The present study was conducted to examine the relationship between urinary incontinence and sleep complaint among a sample of community dwelling older adults 60 years and older in Iran. The results showed that around one-fourth of the sample had urinary incontinence. According to previous studies, urinary incontinence is a common condition among the elderly^29^^-^^32^ and is one of the factors that can greatly affect their quality of life and social interactions^22^. Since the prevalence of urinary incontinence depends on the definition of urinary incontinence that is used, as well as the type and method of the study, its prevalence has been reported to range from 2% to 55%^33^.

Moreover, this present study showed that one-third of the elderly had sleep complaint. This finding is slightly different from previous National Health Survey in Iran, which found around 45% of the elderly had sleep disorder^11^. Similarly, in an Italian study on the elderly, the prevalence of sleep disorder was reported to be 50%^34^. In the United States, the prevalence of some types of sleep disorders in the elderly was even greater and reached about 57%^35^.

In the study conducted on a sample of 656 elderly people 65 years and over in north London, the prevalence of sleep complaint was 44.7%. The study defined sleep complaint was as positive response to a single question namely “Have you had trouble sleeping over the past month?”^36^.

In this present study, the prevalence of sleep complaint was lower than that reported in other studies. This difference may appear to be attributed to the study methods, scales of sleep disorder assessment, and age range of the study samples.

The results of bivariate analysis supported this possibility. Moreover, the results of multiple logistic regression analysis showed a significant relationship between urinary incontinence and sleep complaint after controlling for variables including age, sex, history of hypertension, myocardial infarction, diabetes, vision problems, hearing impairment, and BMI.

The results showing urinary incontinence as the most important factor contributed to sleep complaint are consistent with the finding from an Italian study which found urinary incontinence in the elderly was related to nocturnal sleep disorder and daily sleepiness^19^. Additionally, the results of another study showed sleep disorder in elderly women with urinary incontinence is higher than in elderly women without urinary incontinence^22^.

The urinary incontinence can contribute to sleep complaint in the elderly through several possible mechanisms. 1- Urinary incontinence can directly or indirectly lead to sleep complaint resulting from psychosocial factors, such as social isolation, psychological distress, depression, anxiety, and reduced emotional and social well-being^37^^-^^40^. 2- Concerns about urinating during sleep and fear of being blamed by others can have negative effects on the sleep of an individual and prevent them from falling asleep^41^^,^^42^. 3- Frequent awakening for urination can cause nocturnal sleep disorder^43^. Finally, soaked clothes and mattresses, as well as the unpleasant smell of urine, may decrease the quality of sleep.

Although the current study demonstrated a relationship between urinary incontinence and sleep complaint after adjusting for potential covariates including age, sex, hypertension, myocardial infarction, diabetes, vision problems, hearing impairment, and BMI, it should be addressed that the current study employed a cross sectional design, which precludes causal inferences. Therefore, it is suggested that future studies use a longitudinal deign to gain a better understanding of the causal relationship. Another limitation that should be acknowledged is that data were collected from medical records and therefore might not be completely accurate.

## CONCLUSION

The findings from the present study showing urinary incontinence as a unique predictor of sleep complaint among older adults, imply that effective interventions should be done for controlling urinary incontinence to reduce sleep complaints.
